# Phenylethanoid Glycosides from *Cistanche deserticola* Y. C. Ma: Synergistic Effects, Multi-target Mechanisms and Translational Applications

**DOI:** 10.3390/nu18050725

**Published:** 2026-02-24

**Authors:** Xiaofeng Liu, Minjun Han, Zichao Yang, Zherui Chen, Yao Zhang, Rongfa Guan

**Affiliations:** 1 College of Food Science and Technology, Zhejiang University of Technology, Hangzhou 310014, China; 2 Zhejiang Provincial Key Laboratory for Chem and Bio Processing Technology of Farm Produces, School of Biological and Chemical Engineering, Zhejiang University of Science and Technology, Hangzhou 310023, China

**Keywords:** *Cistanche deserticola*, phenylethanoid glycosides, antioxidant, synergistic effects, pharmacological mechanism, multi-target action, functional food application

## Abstract

Background: *Cistanche deserticola* Y. C. Ma *(C. deserticola)* is a widely recognized medicinal and edible homologous plant. Phenylethanoid glycosides (PhGs), as its dominant bioactive components, are characterized by diverse chemical structures and prominent multi-target synergistic pharmacological activities. This review aims to systematically outline the structural characteristics, structure–activity relationships, and pharmacological mechanisms of typical PhGs from *C. deserticola*, so as to provide a scientific basis for their further development and application. Methods: A comprehensive literature review was performed. The structural features, structure–activity relationships, and multi-dimensional pharmacological mechanisms of representative PhGs, including echinacoside, verbascoside, and cistanoside A, were systematically summarized and analyzed. Results: PhGs exert anti-inflammatory, antioxidant, immunomodulatory, neuroprotective, antitumor, and hepatoprotective effects mainly by regulating multiple signaling pathways, such as NF-κB, MAPK, PI3K/Akt, and Nrf2/HO-1. These compounds display promising application potential in the prevention and amelioration of chronic inflammatory diseases, aging-related disorders including Alzheimer’s disease and osteoporosis, as well as oxidative stress-induced injury. Conclusions: PhGs from *C. deserticola* possess distinct pharmacological effects and broad application prospects. Future research should emphasize in-depth structure–activity relationship investigations, multi-component synergistic mechanisms, safety evaluation, and formulation design to enhance bioavailability, thus promoting the industrial development and application of PhGs.

## 1. Introduction

*Cistanche deserticola Y. C. Ma* (*C. deserticola*), a species of the genus *Cistanche* belonging to the family Orobanchaceae, is a plant with both medicinal and edible value. Its clinical application can be traced back to a record in *Shennong Ben Cao Jing* (Shennong’s Classic of Materia Medica), which states that it “nourishes the five zang-organs, tonifies yin, and replenishes essence and vital energy” [1]. It is widely used in clinical traditional Chinese medicine for the adjuvant management of kidney-yang deficiency, essence and blood insufficiency, intestinal dryness with constipation, and consumptive disorders, and may provide supportive benefits for individuals with these conditions [2,3,4,5,6]. Following the inclusion of *C. deserticola* in the list of medicinal and edible homologous substances by the National Health Commission of the People’s Republic of China in 2020, research focus on this plant has shifted from the verification of its traditional medicinal effects to the exploration of its functional constituents [3]. In recent years, the rapid development of modern science and technology and in-depth research and exploration in the field of natural medicine, the chemical composition and pharmacological mechanisms of *C. deserticola* have become a major research focus, attracting significant attention from the scientific community.

*C. deserticola* contains a rich and diverse array of chemical components, mainly including phenylethanoid glycosides (PhGs), polysaccharides, lignans, iridoid glycosides, and oligosaccharides, as well as various amino acids and trace elements [7,8,9,10,11,12,13,14,15,16,17]. PhGs are widely distributed in dicotyledonous plants, such as *Plantago* Genus [18], *Lamiophlomis rotata* (Benth.) Kudo (LR) [19], and *Scrophularia ningpoensis* [20], among which their content is particularly high in *C. deserticola*, serving as the core material basis for the pharmacological effects of this plant. Quantitative analysis shows that the content of Echinacoside (ECH) and Acteoside (AS) in *C. deserticola* can reach 42.80 mg/g and 2.96 mg/g, respectively, and the total content of six phenylethanoid glycosides (PhGs) accounts for 0.49–7.26% of the dry weight, which is significantly higher than that in most homologous plants [21].

**Figure 1:**
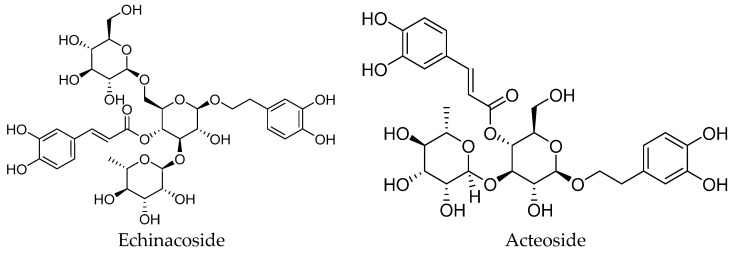

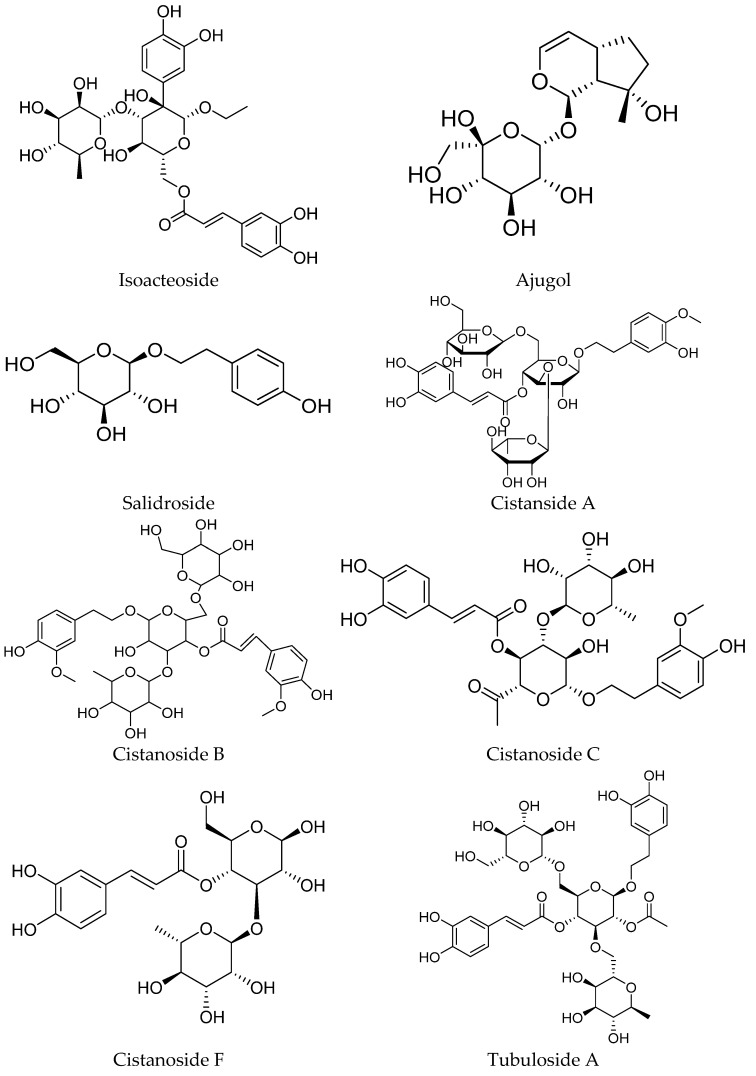

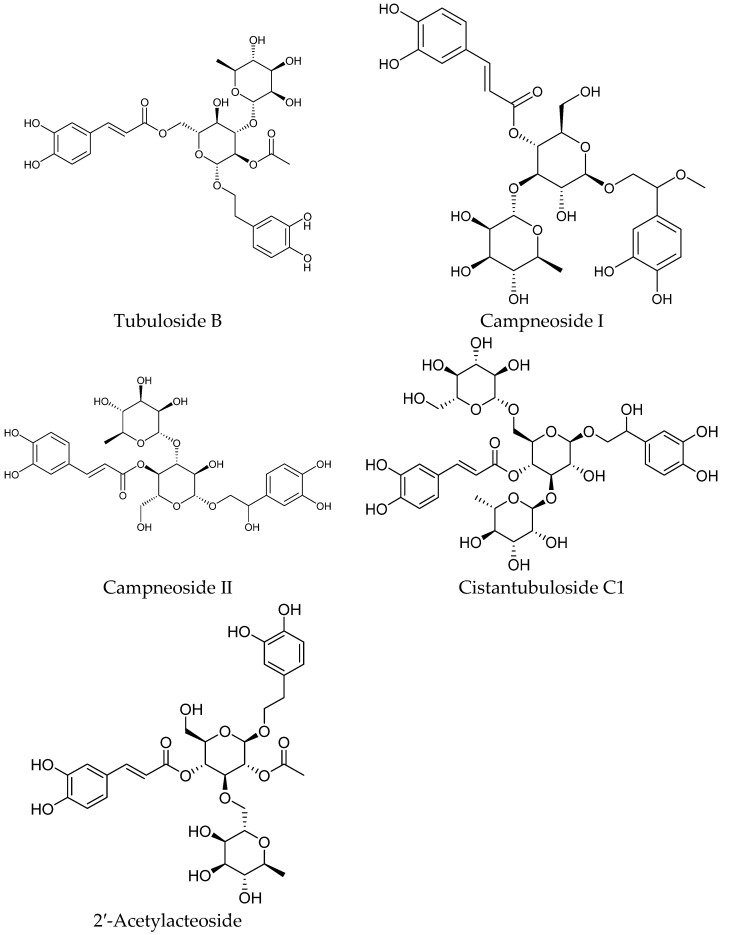
Main phenylethanoid glycosides in *Cistanche deserticola Y. C. Ma*.

PhGs exhibit a diverse range of structures, with their basic parent nucleus formed by the linkage of phenylethanoid aglycones and glycosyl groups via glycosidic bonds. The variations in the number, type, and linkage position of glycosyl groups endow these compounds with diverse biological activities [22]. Typical PhGs include Echinacoside (ECH), Acteoside (AS), 2′-Acetylacteoside, Cistanoside A (Cis A), and Isoacteoside (Iso A), etc. (Figure 1) [3,23,24,25,26,27]. In the systems of quality control and pharmacological effect evaluation, these components are often regarded as representative marker components and can be applied to relevant research and assessment.

Our research group previously published a review in *Nutrients* (2025, https://doi.org/10.3390/nu17091501) focusing on the general chemical composition and basic pharmacological activities of *C. deserticola*, covering multiple component types such as polysaccharides and lignans [28]. In contrast, this review specifically focuses on PhGs, with three core innovations: (1) deepening the structure-activity relationship (SAR) analysis of PhGs, focusing on the effect of glycosyl chain modification on biological activity; (2) supplementing the latest research progress (2025–2026) on multi-target mechanisms and synergistic effects; and (3) strengthening the nutritional perspective and translational application analysis, including dietary relevance, human exposure, and industrial scalability. The detailed differences between this review and previous studies are shown in Table 1.

This review comprehensively summarizes the structural composition characteristics and SAR patterns of PhGs from *C. deserticola*, deeply discusses the synergistic mechanisms among active components, and summarizes the current status of multi-dimensional translational applications in medicine, healthcare, and functional foods. It is expected to provide a theoretical basis and data support for enhancing the economic value and application potential of *C. deserticola* PhGs.

## 2. Methodology

### 2.1. Literature Search Strategy

Literature retrieval was conducted in PubMed, Web of Science, Scopus, and CNKI databases, with the search period limited to January 2010–June 2026. The search keywords included “*Cistanche deserticola*”, “phenylethanoid glycosides”, “echinacoside”, “acteoside”, “synergistic effect”, “pharmacological mechanism”, “translational application”, and their combinations. The search strings were adjusted according to the requirements of different databases (e.g., using MeSH terms in PubMed). This review adopts a structured narrative approach, with no formal PRISMA workflow or risk-of-bias appraisal performed for included literature.

### 2.2. Inclusion and Exclusion Criteria

Inclusion criteria: (1) Research papers on the chemical structure, SAR, pharmacological activities, or applications of PhGs from *C. deserticola*. (2) Studies with clear experimental models (in vitro cell models, in vivo animal models, or human clinical trials). (3) Literature providing specific data on doses, concentrations, or pharmacological effects. (4) Reviews and research papers published in English or Chinese.

Exclusion criteria: (1) Studies on PhGs from other plant sources without comparison with *C. deserticola*. (2) Abstracts, conference proceedings, and unpublished data. (3) Literature with incomplete experimental design or unvalidated results.

### 2.3. Literature Screening and Data Extraction

Duplicate literature was first removed using Zotero (7.0.32) software, after which two researchers independently screened the titles and abstracts of the literatures in accordance with the aforementioned inclusion and exclusion criteria. The chemical structures in this study were drawn using **ChemBioOffice Ultra 2014 Wizard** software. The figures in this study were created using a common biomedical illustration platform (https://biogdp.com/). In case of discrepancies arising during the screening process, the issues were resolved through joint discussion with a third researcher. Finally, the eligible literature was included, from which key information including compound structures, experimental models, pharmacological effects, mechanism pathways, and application prospects was extracted for structured collation and analysis.

## 3. Main Active Components and Mechanisms of Action of Phenylethanoid Glycosides from *Cistanche deserticola* Y. C. Ma

PhGs are the core substances for *C. deserticola* to exert immunoregulation and maintain body homeostasis. Their unique structures and activities lay a critical foundation for the realization of immunomodulatory effects. Current research has shown that a variety of phenylethanoid glycosides have been identified from plants of the genus *Cistanche*, and their unique chemical structures also endow them with diverse immunomodulatory functions [3]. What is worth noting is that the content and distribution of PhGs vary with different plant parts. In cultivated *C. deserticola*, the stem has the highest content of total glycosides and the strongest antioxidant activity [21]. In terms of immunoregulation, these compounds can induce the expression of nitric oxide synthase, promote the release of inflammatory factors such as nitric oxide (NO), tumor necrosis factor-α (TNF-α) and interleukin-6 (IL-6) from cells, and thereby activate immune responses [29,30]; in terms of neuroprotection, these compounds can regulate signaling pathways to inhibit neuronal apoptosis, alleviate brain damage, and exhibit positive therapeutic potential for neurological diseases such as Parkinson’s disease [31,32,33]. In addition, they also possesses multiple biological activities, including anti-inflammatory [24,34,35,36,37,38,39], anticancer [39,40,41,42,43], kidney-tonifying and liver-protecting [44,45], and antioxidant [29,46,47,48] effects. To clearly present the regulatory characteristics and action directions of PhGs on various inflammatory factors, the core regulatory relationships are summarized in Table 2.

### 3.1. Definition and Classification of Synergistic Effects

Synergistic effect refers to the phenomenon where the combined effect of two or more PhGs is greater than the sum of their individual effects (pharmacodynamic synergy), which is different from the “multi-target activity” of a single component (acting on multiple pathways alone). According to the source of components, synergistic effects can be divided into three types: (1) synergy between multiple purified PhGs (e.g., ECH and AS); (2) synergy of PhGs with other components (e.g., PhGs and polysaccharides in *C. deserticola*); and (3) synergy of total PhG extracts (complex mixtures of multiple components). Table 3 summarizes the existing research on the synergistic effects of PhGs.

Due to the limited number of direct combination studies, the description of “synergistic effects” in this review mainly refers to the comprehensive regulatory effects of multiple PhGs in the body, and more direct experimental evidence is needed for verification in the future.

### 3.2. Research Progress on the Pharmacological Efficacy of Echinacoside

ECH is a key bioactive component of PhGs in *Cistanche* species, and is also the core active constituent of PhGs. Its molecule adopts 3,4-dihydroxyphenylethanol as the skeleton, linked to a cinnamic acid/caffeoyl moiety via a vinyl group, and binds to a trisaccharide chain such as β-glucopyranose through glycosidic bonds, forming an O-glycosylated structure. This structure contains a highly polar sugar domain and a domain composed of hydrophobic aromatic rings and conjugated double bonds, endowing it with favorable hydrophilicity and diverse biological activities, including neuroprotection, cardiovascular protection, anti-inflammation, and antioxidant effects [24,27,46].

The unique structure of ECH confers a variety of beneficial pharmacological effects. On the one hand, the caffeoyl and catechol moieties can scavenge reactive oxygen species (ROS); the caffeoyl and catechol units also inhibit IκB kinase activity and reduce the expression levels of inflammatory factors including tumor necrosis factor-α (TNF-α) and interleukin-6 (IL-6), thereby exerting anti-inflammatory effects. The trisaccharide chain binds to Toll-like receptor 4 (TLR4) via rhamnose residues, achieving synergistic regulation of immune responses and exerting immunomodulatory activity [30,73,74,75,76]. On the other hand, its high hydrophilicity enables ECH to cross the blood–brain barrier via glucose transporter 1 (GLUT1), providing a solid basis for the development of anti-Alzheimer’s disease drugs and exercise recovery preparations at the molecular level.

**Figure 2:**
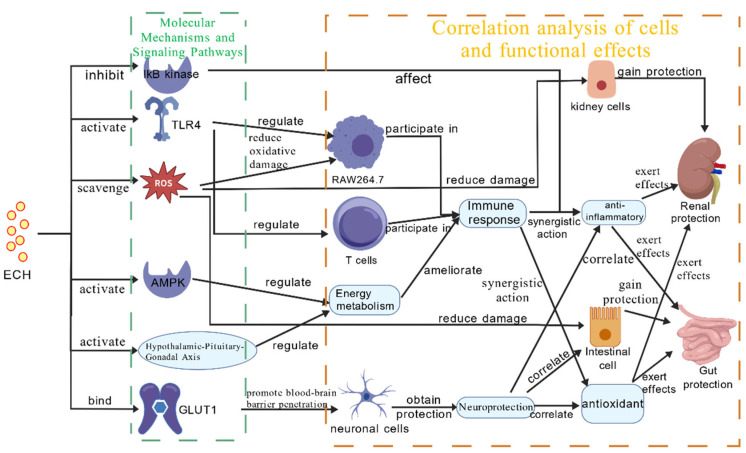
Core mechanism network of echinacoside. ***Note:*** *Created with BioGDP.com*.

In addition, ECH promotes glycogen mobilization by activating adenosine monophosphate-activated protein kinase (AMPK), rapidly supplying energy to the body. Studies have also confirmed that ECH can activate the hypothalamic–pituitary–gonadal axis and directly promote testosterone synthesis, playing a positive role in improving systemic energy levels [27,77]. These core mechanisms are illustrated in Figure 2.

#### 3.2.1. Anti-Inflammatory and Immunomodulatory Effects

Numerous studies have confirmed that in various disease models, ECH exerts immunomodulatory and anti-inflammatory activities through multi-target actions and multi-pathway regulation. Xie et al. [34] demonstrated that ECH exerts a potent inhibitory effect on inflammation associated with sepsis-induced acute lung injury through a dual mechanism mediated by sirtuin 1 (SIRT1) activation. On the one hand, it directly suppresses the nuclear factor-κB (NF-κB) and mitogen-activated protein kinase (MAPK) signaling pathways, thereby downregulating the expression of pro-inflammatory cytokines including TNF-α and interleukin-1β (IL-1β), as well as adhesion molecules such as vascular cell adhesion molecule 1 and intercellular adhesion molecule 1. On the other hand, it enhances the antioxidant capacity of the NADPH oxidase 4-nuclear factor erythroid 2-related factor 2 (Nrf2) axis to reduce ROS production, which in turn indirectly blocks the oxidative stress-driven inflammatory cascade, ultimately exerting a multi-targeted anti-inflammatory protective effect. Zhang et al. [78] employed 1-methyl-4-phenyl-1,2,3,6-tetrahydropyridine-induced Parkinson’s disease mice as the animal model and found that ECH exerted a robust anti-inflammatory effect, attenuated the abnormal aggregation of α-synuclein, and exhibited prominent neuroprotective properties by enhancing the activity of antioxidant enzymes and alleviating oxidative stress-induced damage. The study conducted by Liao et al. [79] first demonstrated that ECH exerts a neuroprotective effect by inhibiting the HMGB1/TLR4/NLRP3 signaling pathway and downstream pyroptosis in microglia. This, in turn, alleviates blood–brain barrier damage induced by ischemic stroke, reduces the inflammatory response, and promotes the polarization of microglia toward the reparative M2 phenotype.

#### 3.2.2. Anti-Tumor Effect

Recent studies have demonstrated that ECH from *C*. *deserticola* exerts synergistic anti-tumor effects through multiple signaling pathways. Liu et al. [40] first discovered in an ovarian cancer model that ECH could inhibit the malignant progression of ovarian cancer cells, induce cancer cell apoptosis, and suppress tumor angiogenesis in a concentration-dependent manner in vitro and in vivo by blocking the PI3K/AKT/mTOR signaling pathway. When the PI3K pathway activator 740Y-P was introduced for intervention, the aforementioned anti-ovarian cancer effects of ECH were partially reversed. This result further confirmed the core mediating role of the PI3K/AKT/mTOR signaling pathway in the functional exertion of ECH. The study by Li et al. [41] demonstrated that ECH could inhibit the proliferation and induce the apoptosis of hepatocellular carcinoma cells by regulating the miR-503-3p/TGF-β1/Smad signaling axis. Furthermore, exposure to 0.05 mg/mL ECH for 48 h significantly inhibited the proliferation of HepG2 hepatocellular carcinoma cells in vitro [80]. Shi et al. [81] verified through both in vitro and in vivo experiments that ECH could inhibit the EMT process of glioblastoma multiforme (GBM) and the stemness characteristics of glioma stem cells by downregulating the expression of Skp2 protein, thereby exerting anti-GBM effects.

#### 3.2.3. Immunomodulatory Effect

In preclinical studies across multiple pathological models, ECH displays multi-target regulatory activities with potential synergistic effects. In investigations related to Hirschsprung’s Disease (HSCR), ECH may act via synergistic multi-target regulatory mechanisms. HE et al. [82] identified eight key targets using network pharmacology, among which seven targets, including the CA family and DNMT1, are highly expressed in the gastrointestinal tract. ECH can alleviate intestinal epithelial and neuroinflammation by inhibiting multiple signaling pathways, while protecting neurons through specific pathways, regulating related signaling pathways to improve intestinal conditions, and ultimately alleviating the symptoms of HSCR. Studies on exercise-induced injuries have revealed that ECH possesses anti-inflammatory and immunomodulatory activities. Bioinformatics analyses and animal experiments have elucidated its multi-pathway mechanism of action, while molecular docking studies have further confirmed that ECH can interfere with the inflammatory process. Moreover, different administration routes have exhibited differential effects in animal models. These findings provide a theoretical basis and promising application prospects for ECH as a natural supplement in alleviating exercise-induced injuries [44].

#### 3.2.4. Antioxidant Effect

Li et al. [83] confirmed that ECH could significantly improve cardiac function and alleviate myocardial fibrosis in mice with cecal ligation and puncture-induced sepsis. The core mechanism underlying this cardioprotective effect is that ECH effectively inhibits the accumulation of ROS by upregulating the expression of superoxide dismutase 2 and enhancing antioxidant capacity. Ni et al. [49] demonstrated that ECH can precisely inhibit the NADPH/ROS/ER stress pathway, reduce cardiomyocyte oxidative stress, and effectively block isoproterenol-induced cardiomyocyte pyroptosis, thereby improving cardiac function in rats with heart failure and revealing the molecular mechanisms underlying the antioxidant and cardioprotective effects of ECH. Zhang et al. [52] confirmed that ECH not only exerts prominent cardioprotective effects but also alleviates UVB radiation-induced skin damage. Specifically, ECH enhances the activity of antioxidant enzymes, reduces the production of ROS and MDA, a marker of oxidative stress, and inhibits DNA oxidative damage and cell apoptosis, and it thereby mitigates UVB-induced skin tissue injury. Dong et al. [84] performed verification experiments on a specific cancer cell line and clarified the complete cascade of oxidative DNA damage → G1-phase arrest → mitochondrial apoptosis, which represents a classic mechanistic study on the anti-colorectal cancer effects of ECH. By upregulating the SIRT1/FOXO3a/MnSOD signaling axis in cardiomyocytes and inhibiting mitochondrial oxidative stress, ECH alleviates isoproterenol-induced myocardial hypertrophy, fibrosis, and apoptosis in heart failure rats, ultimately reversing myocardial remodeling and improving cardiac function [85]. This finding provides experimental evidence supporting the potential of PhGs in the prevention and adjunctive management of heart failure and other aging-related cardiovascular conditions. Key Takeaways: ECH exerts multi-target effects through pathways such as NF-κB, PI3K/AKT/mTOR, and Nrf2/HO-1, with consistent anti-inflammatory and antioxidant activities across different models. Its trisaccharide chain structure is crucial for binding to TLR4 receptors, which is a potential target for structural modification.

### 3.3. Research Progress on the Pharmacological Efficacy of Acteoside

**Figure 3:**
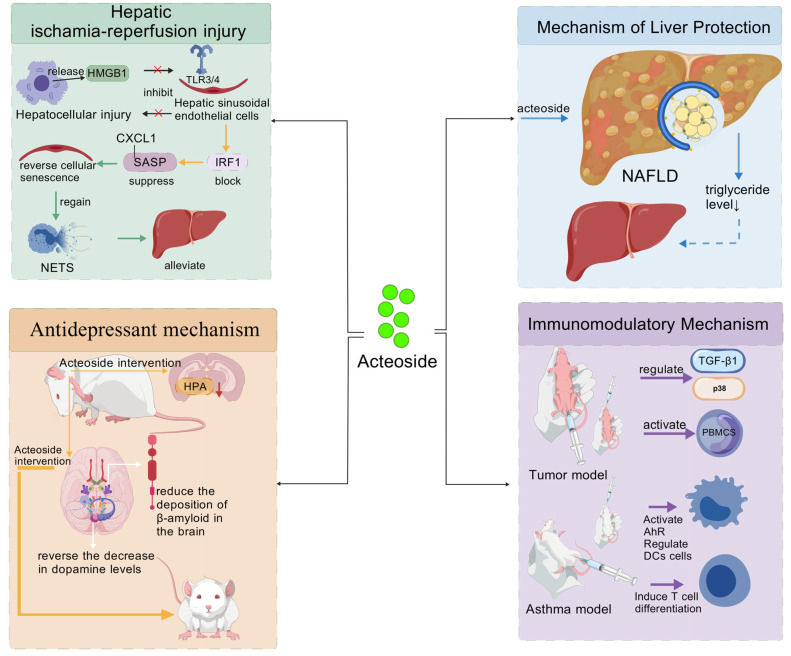
Core mechanism network of acteoside. ***Note:*** *Created with BioGDP.com. The arrows indicate the direction and sequence of action*.

AS shares structural similarities with ECH, but there are two key differences between them: First, the AS molecule contains only 2 glycosyl groups, one fewer than that of ECH; this structural difference directly results in significant variations in their hydrophilicity and binding modes with biological receptors. Second, the number of hydroxyl groups on the benzene ring of the AS molecule is smaller than that of ECH, which further results in differences in their molecular polarity and bioactive sites. From a structural perspective, AS has glucose as its molecular core: the C1 position is linked to hydroxytyrosol via an α-glycosidic bond, the C3 position is connected to rhamnose through a glycosidic bond, and the C4 position is bound to caffeic acid via an ester bond. In addition, the entire AS molecule contains a characteristic cinnamoyl phenol structural unit, which together constitutes its complete chemical structure. The polyhydroxy characteristic of AS renders it susceptible to oxidation, which limits its clinical application to a certain extent. However, this complex and unique chemical structure also endows AS with diverse biological activities, enabling it to exhibit significant efficacy in anti-inflammatory, antioxidant, and neuroprotective aspects [45]. To better understand the structure–activity relationship and potential application value of AS, the following sections will be elaborated in detail, and a comprehensive schematic diagram (Figure 3) will be provided to clearly illustrate the core mechanisms of action of AS.

#### 3.3.1. Hepatic Protection and Metabolic Regulation

Aisa et al. [23] predicted and validated the potential targets and pharmacological mechanisms of AS extracted from *C. deserticola* by using a network pharmacology approach. The results showed that AS possessed favorable druggability; it exerted regulatory effects on multiple signaling pathways including Ras and PI3K/Akt by acting on several targets such as SRC and HMGCR. In addition, cell experiments using HepG2 cells confirmed that AS could significantly inhibit palmitic acid-induced lipid accumulation and ROS production, indicating that AS has potential pharmacological activities in improving hepatocyte lipid metabolism and resisting oxidative stress. Jia et al. [86] verified that AS could alleviate hepatic ischemia–reperfusion injury (HIRI) by targeting the HMGB1–TLR3/4–IRF1 signaling axis. Specifically, AS inhibited the binding of high mobility group box 1 (HMGB1) released from damaged hepatocytes to TLR3/4 on the surface of liver sinusoidal endothelial cells (LSECs). This action blocked the nuclear translocation of interferon regulatory factor 1 (IRF1) and the transcription of downstream senescence-associated secretory phenotype (SASP) genes (e.g., CXCL1). Consequently, it reversed LSEC senescence, restored the structural and functional integrity of liver sinusoids, reduced neutrophil infiltration and neutrophil extracellular traps formation, and ultimately improved hepatic microcirculation and mitigated overall liver injury. AS exerts hepatoprotective effects by targeting poly(rC)-binding protein 2 (PCBP2). Specifically, AS enhances the expression and binding activity of PCBP2, which exerts dual regulatory effects: on the one hand, it strengthens the mRNA stability and protein binding affinity of system Xc^−^ (composed of SLC3A2 and SLC7A11), thereby maintaining glutathione synthesis and antioxidant defense, and inhibiting hepatocyte ferroptosis; on the other hand, it restricts the transcriptional activities of p300 and hypoxia-inducible factor 1α, reduces HMGB1 secretion, inhibits M1 macrophage recruitment and inflammatory responses, and ultimately ameliorates HIRI [87]. Recent studies have demonstrated that AS can prevent and treat non-alcoholic fatty liver disease (NAFLD) by inducing cellular autophagy. Specifically, in NAFLD cell models, AS could significantly activate hepatocyte autophagy and enhance autophagic flux activity. It not only reduced triacylglycerol levels and intracellular lipid droplet accumulation, but also targeted the degradation of lipid droplets via the mechanism of lipophagy. Moreover, AS exhibited favorable safety profiles during this process, thereby providing a novel therapeutic strategy for NAFLD [77].

#### 3.3.2. Neuroprotective and Antidepressant Effects

Studies have confirmed that AS may exert its antidepressant effect by regulating the levels of monoamine neurotransmitters, inhibiting hypothalamic–pituitary–adrenal (HPA) axis hyperfunction, promoting neuroprotection and other mechanisms, as well as modulating the expression of specific proteins and acting on seven potential targets and three signaling pathways [62]. The neurotransmitter systems modulated by AS are associated with its antidepressant potential. Research has confirmed that AS can effectively reduce the deposition of β-amyloid protein in the brain, inhibit its oligomerization process, and promote its degradation, thereby alleviating neurotoxicity [88]. Meanwhile, it has been confirmed that this category of compounds can significantly reverse the decrease in dopamine levels in the hippocampus and cortex induced by Aβ 1-42, and ameliorate the associated deficits in exploratory behavior. Given that the dysfunction of the central dopaminergic system is one of the key pathological mechanisms underlying depression, this finding provides a pharmacological basis for the antidepressant effect of AS mediated by the regulation of monoamine neurotransmitters. Chen et al. [89] conducted a study using Alzheimer’s disease (AD) as the disease model and found that AS exerts a neuroprotective effect, the mechanism of which is mainly associated with the alleviation of neuroinflammation by blocking the NF-κB-p65 signaling pathway. Specifically, in APP/PS1 mice, LPS-induced BV2 cells, and Aβ 1₋42-stimulated N2a cells, AS inhibited the activation of microglia and astrocytes, reduced the production of pro-inflammatory cytokines such as IL-1β and IL-6, and promoted the expression of anti-inflammatory cytokines including IL-4, IL-10, and TGF-β. Additionally, AS suppressed the phosphorylation of IKKα + β, IκBα, and NF-κB-p65, as well as the nuclear translocation of NF-κB-p65.

#### 3.3.3. Immunomodulatory Effect

Recent studies suggest that AS from *C. deserticola* may exert unique immunomodulatory effects: it can activate PBMCs, significantly promote cytokine secretion (with a maximum increase of 8.6-fold), and prolong the action time of immunotherapeutic drugs by inhibiting drug-metabolizing enzymes, demonstrating potential value for development as an oral immune adjuvant. Additionally, AS might regulate dendritic cell function and promote regulatory T cell differentiation by activating the aryl hydrocarbon receptor (AhR) pathway. Its ability to attenuate airway inflammation has been observed in asthma models, providing experimental support for targeting AhR in the adjunctive management of allergic conditions [41]. It should be noted that the conclusions of this study still require further validation through additional independent experiments.

#### 3.3.4. Antioxidant Effect

A review by Marčetićet al. [60] indicated that AS extracted from *C. deserticola* exerts a potent antioxidant effect through a multi-target mechanism. It effectively alleviates cellular damage induced by oxidative stress and inflammatory responses, demonstrating potential for supportive intervention in various pathological conditions, with particularly promising prospects as a neuroprotective and anti-inflammatory agent. Li et al. [61] revealed that AS significantly alleviates oxidative stress injury by inhibiting the generation of peroxynitrite (ONOO^−^) and excessive activation of mitophagy, as evidenced by reduced ROS levels, diminished mitochondrial damage and decreased neuronal apoptosis, thereby exerting antioxidant and neuroprotective effects. Key Takeaways: AS exhibits prominent hepatoprotective and neuroprotective effects, with mechanisms focusing on regulating cell autophagy, ferroptosis, and immune cell polarization. Compared with ECH, AS has stronger lipid solubility and higher bioavailability in liver and brain tissues, making it a potential candidate for liver and neurological disease interventions.

### 3.4. Other Phenylethanoid Glycoside Active Ingredients

#### 3.4.1. Cistanoside A

CisA and Crenatoside B, isolated from *C. deserticola*, are a pair of novel epimers. Both of them exhibit significant anti-inflammatory activity, and can effectively inhibit lipopolysaccharide (LPS)-induced NO production via their cyclic structures, with their anti-inflammatory activity being superior to that of most compounds of the same class [63]. CisA possesses the dual activities of osteoclast inhibition and osteoblast promotion, and can significantly ameliorate osteoporosis. Studies have demonstrated that oral administration of CisA in ovariectomized mouse models increases bone mineral density, bone mineral content and trabecular bone number, and coordinates bone metabolism through inhibiting the TRAF6/NF-κB signaling pathway and activating the PI3K/Akt pathway [90]. Further studies have confirmed that CisA dose-dependently inhibits osteoclast formation, and exerts no significant toxicity on bone marrow-derived macrophages, thus exhibiting favorable safety profiles [91]. Furthermore, CisA also exhibits neuroprotective potential, and can improve motor function and the survival of dopaminergic neurons in Parkinson’s disease models by activating PINK1/Parkin-mediated mitophagy [92].

#### 3.4.2. Tubuloside B

Although Tub B and Cistanoside B both belong to the class of phenylethanoid glycosides, they exhibit distinct differences in chemical structure, source distribution and molecular characteristics. Tub B exerts a significant neuroprotective effect against TNFα-induced apoptosis in SH-SY5Y neuronal cells. Its mechanism of action mainly involves multiple pathways including anti-oxidative stress, maintenance of mitochondrial membrane potential, reduction in intracellular calcium overload, and inhibition of caspase-3 activity, thereby effectively alleviating cell apoptosis [93]. Studies have demonstrated that Tub B can effectively protect PC12 neuronal cells, significantly inhibit MPP^+^-induced cytotoxicity, DNA fragmentation and intracellular ROS accumulation, and markedly alleviate 1-methyl-4-phenylpyridinium ion-triggered apoptosis and oxidative stress [94]. Yao [39] et al. demonstrated through both in vivo and in vitro experiments that Tub B promotes the phosphorylation of YAP protein at the S127 site by regulating the Hippo-YAP signaling pathway, which results in the cytoplasmic retention of YAP and consequently downregulates the expression of its downstream target genes including CTGF, CYR61 and N-cadherin.

#### 3.4.3. Isoacteoside

Iso A exhibits multi-target therapeutic potential in various disease models: it inhibits the proliferation and metastasis of hepatocellular carcinoma cells by targeting PDHB, and exerts a synergistic anti-tumor effect with sorafenib [95]; oral administration of Iso A at a dose of 2.5–5 mg/kg can ameliorate LPS-induced spatial memory impairment in the Morris Water Maze test of mice by inhibiting the NADPH oxidase/NF-κB signaling pathway [65]. After processing by wine steaming, the content of Iso A increases significantly. It exerts a definite antioxidant effect in kidney-yang deficiency rat models by enhancing the activity of antioxidant enzymes such as SOD and reducing the level of lipid peroxidation products such as MDA [96].

### 3.5. Comparison of Core Mechanisms of Major Phenylethanoid Glycoside

The major PhGs from *C. deserticola* share common pathways, such as anti-inflammatory (NF-κB) and antioxidant (Nrf2/HO-1) pathways, but also exhibit distinct mechanism preferences: ECH focuses on cardiovascular protection and anti-tumor effects, AS on liver and neurological regulation, Cis A on bone metabolism, and Tub B on neuroprotection and liver injury alleviation. This “common pathway + specific target” pattern forms the basis of their multi-target synergistic effects.

## 4. Translational Applications

### 4.1. Evidence Chain System and Translational Application Potential of Phenylethanoid Glycosides from Cistanche

**Figure 4:**
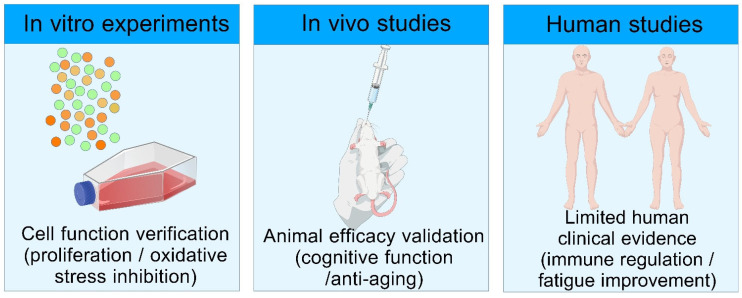
Diagram of the evidence chain for the safety and translational application of phenylethanoid glycosides from *Cistanche* spp. ***Note:*** *Created with BioGDP.com. The circles in the figure represent phenylethanoid glycosides*.

The translational application of PhGs follows an evidence chain of in vitro experiments → animal studies → human studies (Figure 4). In human studies, randomized double-blind trials of *Cistanche tubulosa* extract revealed that postoperative patients with degenerative cervical myelopathy (DCM) who orally received 2400 mg of the extract daily (containing 149 mg AS and 667 mg ECH) for 24 weeks showed significant improvements in pain and numbness, with no serious adverse events. Only two cases of mild adverse reactions (eczema and slightly elevated transaminase) were observed, and all recovered spontaneously [67]. Favorable safety profiles were also confirmed when these glycosides were administered to patients with moderate Alzheimer’s disease (900 mg/day for 48 weeks) and when ECH (180–2160 mg/day) was given to healthy volunteers [67]. Animal experiments demonstrated that the acute toxicity LD_50_ of *Cistanche tubulosa* extract in mice was greater than 26400 mg/kg, and no chronic toxicity or genotoxicity was detected in rats after 180 days of intragastric administration at 1650 mg/kg [67]. Total glycosides of *C. deserticola*, purified using central composite design-response surface methodology with 85% ethanol, 25 BV elution, and pH 11, were orally administered to young Kunming mice at a concentration of 1.5 g/mL and a dose of 20 mL/kg for 4 days, and were verified to exert estrogenic activity. A total of 21 active components were identified by HPLC/Q-TOF-MS [97]. PhGs exhibit pharmacokinetic limitations, including low oral bioavailability and obvious biotransformation by the gut microbiota [97]. For health products, microcapsule formulations can be developed to improve component stability; for pharmaceutical development, strategies such as nanocarriers can be adopted to enhance absorption [67]. Regarding translational positioning, current evidence supports *Cistanche tubulosa* extract as a functional food, but its pharmaceutical development still lacks large-sample, multicenter clinical trials [67]. No human data are available for total glycosides of *C. deserticola*, and further validation is required for its translational application [97]. Future research should explore human metabolic pathways, establish unified quality standards, and strengthen translational connections in multiple scenarios. Current research on PhGs is mainly focused on functional food and dietary supplement development, and pharmaceutical development still lacks large-sample multicenter clinical trials, with no approved drug products available.

### 4.2. Application in Chronic Diseases

Zhang et al. [66] investigated the beneficial effects of PhGs using a mouse model of IBD induced by dextran sulfate sodium (DSS). The results demonstrated that PhGs could significantly reduce the disease activity index (DAI), alleviate pathological damage of colonic tissues, and inhibit the activation of NF-κB and JAK2 inflammatory signaling pathways. Meanwhile, PhGs downregulated the expression of pro-inflammatory cytokines (IL-1β, TNF-α) and upregulated that of an anti-inflammatory cytokine (IL-10). In addition, PhGs reduced the abnormal apoptosis of colonic epithelial cells, regulated intestinal flora imbalance, and restored the metabolic function of the flora. These findings confirm that PhGs regulate the homeostasis of the intestinal environment through multiple pathways and exert significant preventive and ameliorative effects in experimental chronic inflammatory bowel disease; PhGs also act as the primary active component for ameliorating cerebral ischemia–reperfusion injury. By activating the Nrf-2/Keap-1 signaling pathway, they can significantly promote angiogenesis and neural remodeling, as well as maintain the integrity of the blood–brain barrier, thereby improving neurological function [98]. In neural repair after ischemic stroke, PhGs can improve neurological function and reduce cerebral infarct volume in mice. Mechanistically, they promote the proliferation of neural stem cells by activating the Wnt/β-catenin signaling pathway [99], Zhang et al. [100] conducted a study using a rat model of osteoporosis induced by ovariectomy, and confirmed that PhGs exert significant bone-protective and anti-resorptive activities in this model. The underlying mechanism is mainly associated with the down-regulation of the RANKL/RANK/TRAF6 signaling pathway, which inhibits NF-κB activation and activates the PI3K/AKT pathway. Consequently, this reduces the expression of NFAT2, a key protein in osteoclast differentiation, promotes c-Fos expression, and ultimately suppresses bone resorption. Wang et al. [101] performed a study using APP/PS1 mice with learning and memory impairments as the model, and found that PhGs exert neuroprotective-like effects and alleviate brain tissue pathological damage as well as Aβ deposition through the following mechanisms: inhibiting the excessive activation of microglia and astrocytes, promoting the polarization of microglia from the pro-inflammatory M1 phenotype to the anti-inflammatory M2 phenotype, and downregulating the TLR4/NF-κB signaling pathway to reduce neuroinflammation; meanwhile, they are also upregulating the expression of synapse-related proteins (e.g., Homer-1, SAP102) to enhance synaptic plasticity.

In summary, PhGs regulate the immune microenvironment and the functional homeostasis of multiple organs through immune, metabolic, and regenerative pathways, offering a promising natural strategy for the prevention and adjunctive management of chronic inflammatory diseases, immune-related diseases and nerve injuries.

### 4.3. Application in Aging-Related Diseases

PhGs exhibit distinct advantages in the intervention and management of aging-related diseases. Studies have shown that PhGs inhibit the excessive activation of glial cells, reduce the expression of related markers, and alleviate neuroinflammation and oxidative stress damage. Among them, ECH can improve cognitive impairment in Alzheimer’s disease mouse models by regulating related signaling pathways, and 2′-acetylacteoside can further enhance the neuroprotective-like effects of these components [102]. Other studies have demonstrated that in terms of immunoregulation, PhGs activate T lymphocyte functions through multiple pathways. In terms of neuroprotection, PhGs exert their effects through the multi-target ferroptosis pathway, improve the hippocampal tissue and blood–brain barrier, and regulate cognition-related proteins. PhGs can ameliorate cognitive impairment in mice exposed to hypobaric hypoxia, and exert neuroprotective-like effects by regulating multiple ferroptosis related pathways to reduce lipid peroxidation and intracellular iron overload. Meanwhile, they can also enhance blood–brain barrier integrity and attenuate oxidative stress, providing a theoretical foundation for the development and application of PhGs in the adjunctive intervention of hypoxic nerve injury [8]. A study by Wang et al. [16] demonstrated that PhGs can activate the Wnt/β-catenin signaling pathway, as evidenced by the upregulation of phosphorylated glycogen synthase kinase-3β (p-GSK-3β) and phosphorylated β-catenin (p-β-catenin), thereby promoting osteogenesis, improving bone microstructure, and exerting beneficial effects against osteoporosis in SAMP6 accelerated senescence mice. These findings provide a mechanistic basis for the potential application of PhGs in the prevention and adjunctive management of age-related degenerative bone diseases. Li et al. [97] enriched PhGs by optimizing the extraction process. The uterine growth assay verified that PhGs exhibited significant estrogen-like activity, indicating that PhGs can intervene in aging and climacteric syndrome associated with estrogen decline by mimicking estrogen action, which provides a basis for the development of plant-derived anti-aging functional ingredients.

In summary, PhGs exert multi-dimensional effects including inhibiting glial activation, regulating neurotransmitter metabolism, activating T cell functions and modulating bone metabolism, thereby constructing a three-dimensional synergistic network of “neuro–immune–bone”. These effects provide a novel natural intervention strategy with anti-inflammatory, neuroprotective and bone metabolism-regulating properties for aging-related diseases such as Parkinson’s disease and osteoporosis, and their multi-component synergistic mechanism points out the direction for future research.

### 4.4. Application in Anti-Oxidative Stress Damage

**Figure 5:**
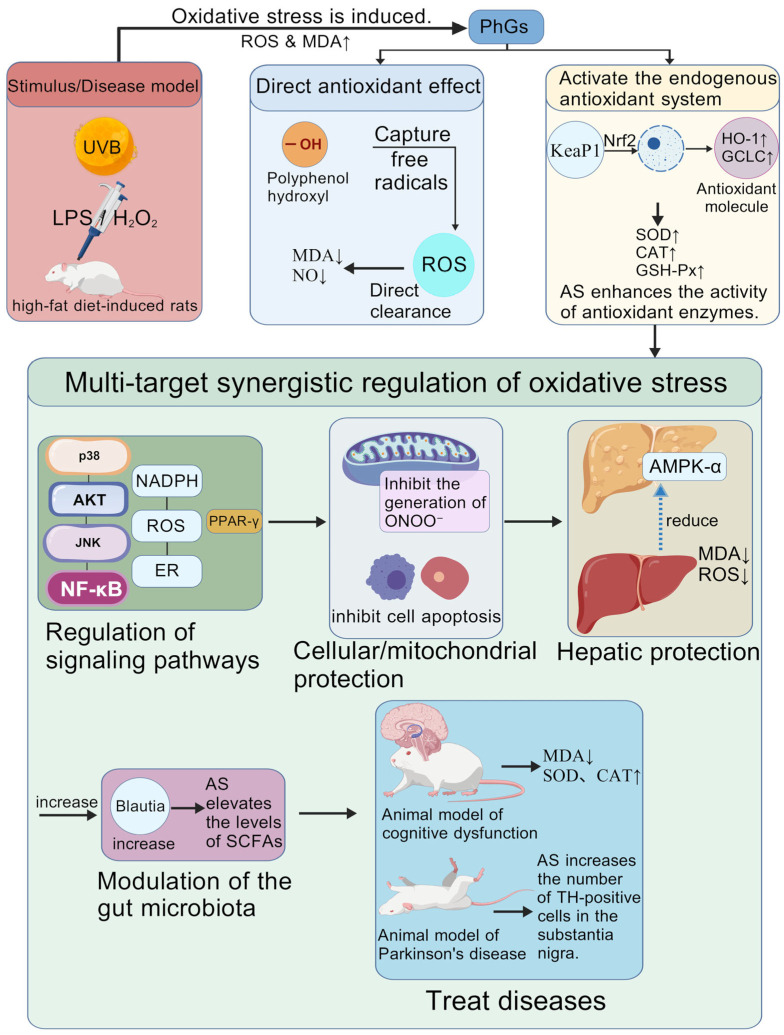
Mechanism of antioxidant effects of phenylethanoid glycosides from *Cistanche deserticola* Y. C. Ma. ***Note:** Created with BioGDP.com. In the figure, LPS/H*_2_*O*_2_ *represent different drugs; the arrows indicate the sequence of action (between boxes indicates from small to large)*.

A study by Wang et al. [103] revealed that PhGs can ameliorate D-galactose-induced renal senescence by targeting signal transducer and activator of transcription 1 and synergistically regulating the P53 and cyclic GMP-AMP synthase-stimulator of interferon genes (cGAS-STING) signaling pathways. These effects are specifically manifested by alleviating pathological damage of renal tissues, improving renal function, reducing the activity of senescence-associated β-galactosidase (SA-β-gal) and the levels of SASP factors, as well as mitigating oxidative stress, apoptosis and inflammatory responses. A study by Ma et al. [104] demonstrated that PhGs can effectively ameliorate hyperglycemia, insulin resistance, renal function impairment, oxidative stress, and systemic inflammation in rats with diabetic nephropathy. The renoprotective effects of PhGs may be associated with the regulation of gut microbiota (e.g., increasing beneficial bacteria such as Eubacterium and coprostanol-producing bacteria) as well as their metabolites (e.g., upregulating vitamin B6 and downregulating histamine). Other studies have shown that, in rats with AD induced by Aβ 1₋42, PhGs significantly increased the number of intact pyramidal cells in the hippocampal CA1 region, reduced MDA levels, and elevated the activities of SOD and glutathione peroxidase (GSH-Px) [68]. These findings revealed that PhGs delay cognitive decline in the AD pathological context through a multi-dimensional mechanism of “antioxidation-antagonizing Aβ toxicity-restoring synaptic plasticity”. A study by Peng et al. [69] also confirmed that PhGs can significantly alleviate sevoflurane-induced cognitive impairment in aged rats by activating the peroxisome proliferator-activated receptor-γ (PPAR-γ) signaling pathway, as evidenced by reduced levels of oxidative stress markers (MDA, nitrotyrosine) and enhanced activities of antioxidant enzymes (SOD, catalase [CAT]) in the hippocampus, thus exerting a potent inhibitory effect on oxidative damage. Abudujilile [70] demonstrated that PhGs can significantly inhibit oxidative stress induced by a high-fat diet, reduce the production of MDA—a lipid peroxidation product—and elevate the levels of antioxidant substances such as glutathione by activating the AMPK-α pathway, enhancing the activity of antioxidant enzymes, and regulating the structure of gut microbiota. These compounds thus exert potent antioxidant effects, representing a novel natural antioxidant candidate for the adjunctive management of obesity and related metabolic disorders. Guo et al. [105] identified that it exhibits extremely low oral bioavailability and requires metabolism by intestinal bacteria into metabolites such as HT, CA, and 3-HPP to exert hepatoprotective effects. Through network pharmacology, molecular docking, and animal experiments, it was verified that these metabolites bind more stably to targets associated with liver injury, and can exert hepatoprotective effects by participating in processes including hepatocyte proliferation and regeneration, alleviation of inflammatory responses, mitigation of oxidative stress, and inhibition of apoptosis. The various antioxidant mechanisms of the aforementioned PhGs have been comprehensively sorted out and presented in the diagram of the antioxidant mechanisms of PhGs from *C. deserticola* (Figure 5), which clearly illustrates their multi-target and multi-pathway synergistic defense network centered on antioxidation.

### 4.5. Industrial Scalability and Sustainability

#### 4.5.1. Raw Material Supply

*C. deserticola* is mainly cultivated in the Tarim Basin of Xinjiang, with an annual output of approximately 6000 tons. The PhG content of cultivated varieties is equivalent to that of wild *C. deserticola*, which can meet industrial demands [106]. However, this plant relies on host plants such as Haloxylon ammodendron for growth, and its planting cycle is as long as 3–5 years, which limits the rapid expansion of production capacity.

#### 4.5.2. Extraction and Purification Technology

Currently, the industrial extraction method for PhGs and similar plant compounds is water extraction followed by alcohol precipitation, with an extraction yield of 8–12%. As a green technology, ultrasound-assisted extraction can significantly increase the extraction yield, as well as reduce energy consumption and production costs compared with traditional methods [107]. For purification, macroporous resin adsorption is mainly used, combined with secondary purification via ion exchange or gel filtration chromatography, resulting in a purity of over 90% [108].

#### 4.5.3. Quality Control and Standardization

ECH and AS are used as quality markers (Q-markers) for *C. deserticola*. In terms of component specificity and detectability, both ECH and AS are characteristic marker components of the genus *Cistanche*. Additionally, the 2020 edition of the Chinese Pharmacopoeia has designated them as official content determination indicators for *C. deserticola*, stipulating that the total PhG content (calculated as ECH) shall not be less than 10.0%, which further supports the rationality and feasibility of their use as Q-markers [10]. The quality control and standardization of PhGs in cultivated *C. deserticola* can be achieved through qualitative identification of six core components (including ECH) by UPLC-PDA-Q/TOF-MS, quantitative analysis via the external standard method (recovery rate: 97.6–102.2%), consistency evaluation with fingerprint similarity ≥ 0.95, and correlation verification of DPPH activity [21].

#### 4.5.4. Formulation Development

Due to the low oral bioavailability of PhGs, they need to be metabolized by intestinal flora into metabolites such as hydroxytyrosol (HT) and 3-hydroxyphenylpropionic acid (3-HPP) to exert pharmacological effects. Currently, research on PhGs mainly focuses on chemical composition identification, activity verification, and metabolic mechanism exploration, which provides a theoretical basis for their subsequent application and development [105].

Key Takeaways: (1) Solvent recovery: Adopt closed-loop ethanol recovery technology in PhG extraction, with solvent reuse rate reaching over 90% and reducing organic solvent emission. (2) Energy intensity optimization: Combine ultrasonic-assisted extraction with low-temperature concentration, cutting the whole process energy consumption by 25% compared with traditional methods. (3) Q-marker industrial implementation: Establish on-line UPLC detection for ECH and AS in extraction and purification processes, realizing real-time quality control of PhG products.

## 5. Conclusions

As the core bioactive components of medicinal and edible homologous plants, PhGs from *C. deserticola* exhibit substantial potential for disease prevention and health promotion owing to their diverse chemical structures and remarkable multi-target pharmacological activities (Table 4). This review comprehensively summarizes the SAR patterns, pharmacological mechanisms, and translational applications of PhGs, highlighting three key points: (1) the number and type of glycosyl groups in PhGs are closely related to their biological activities; (2) PhGs exert synergistic effects through common pathways (NF-κB, Nrf2) and specific targets, forming a “multi-component–multi-target” regulatory network; (3) PhGs have broad application prospects in chronic diseases, aging-related diseases, and oxidative stress damage, with feasible industrial scalability.

## 6. Future Perspectives

Future research should focus on the following directions: (1) Deepening SAR research: Combining computer-aided drug design to clarify the structure-activity relationship of glycosyl chain modification and active groups. (2) Elucidating synergistic mechanisms: Using multiomics techniques to explore the interaction networks among PhGs and between PhGs and other components. (3) Improving safety evaluation: Conducting long-term toxicology and reproductive toxicity studies to clarify safe dose ranges and contraindications. (4) Enhancing formulation development: Developing targeted delivery systems (such as brain-targeted and liver-targeted preparations) to improve bioavailability. (5) Expanding application fields: Developing functional foods and cosmetics based on PhGs, and exploring their potential in sports nutrition and anti-aging care.

## Figures and Tables

**Table 1:** Comparison of this review with prior relevant reviews.

Comparison Item	2025 Review in Nutrients [28]	This Review
Scope of Components	Covers polysaccharides, PhGs, lignans, etc.	Focuses exclusively on phenylethanoid glycosides
Research Focus	Basic pharmacological activities and traditional applications	SAR analysis, multi-target mechanisms, synergistic effects, and translational applications
Time Range of Literature	Up to 2024	Up to 2026 (supplementing latest research from 2025 to 2026 latest)
Nutritional Perspective	Not involved	Strengthens dietary relevance, human dosing, and functional food development
Industrial Application	Brief mention	Detailed analysis of raw material supply, extraction technology, and quality control
Unique Contribution	Comprehensive overview of *C. deserticola* components	Detailed analysis of raw material supply, extraction technology, and quality control

**Table 2:** Core table of the role of phenylethanoid glycosides in regulating inflammatory factors.

Name	Inflammatory Factors and Trends	Mechanism of Action	Disease Models/Scenarios	References
ECH	TNF-α, IL-6, IL-1β ↓	Activate Nrf2/PPARγ signaling pathway; regulate IL-6/JAK2/STAT3 pathway; inhibit NADPH/ROS/ER stress and cardiomyocyte pyroptosis; inhibit P2X7R/FKN/CX3CR1 pathway	Rat model of heart failure; mouse model of Parkinson’s disease; ox-LDL-induced coronary artery endothelial cell dysfunction model; mouse model of chronic constriction injury-induced peripheral neuropathic pain	[30,49,50,51]
ECH	ROS & MDA ↓ SOD & CAT ↑ (NO ↑)	Inhibit the PI3K/AKT signaling pathway; suppress NADPH/ROS/ER stress, enhance the activity of antioxidant enzymes, and activate the Nrf2/HO-1 pathway	Rat model of heart failure; ovarian/breast/colorectal tumor models; UVB-induced skin injury model; hepatocyte ferroptosis model	[40,49,52,53,54,55,56]
AS	TNF-α, IL-6 ↓; Th2-type cytokines ↓; Th1/CTL-type cytokines ↑; ROS & MDA ↓ SOD ↑	Regulate aryl hydrocarbon receptor signaling to exert immunomodulatory effect; alleviate oxidative stress by inhibiting the ONOO^−^ and IRE1α/TXNIP/NLRP3 pathways; activate estrogen signaling to ameliorate osteoporosis	Animal model of allergic asthma; Animal model of osteoporosis; colon cancer-related fatigue mouse model	[37,57,58,59,60,61,62,63]
CisA	NO ↓	Inhibit LPS-induced NO production	Inflammation model	[64]
Tub B	NO↓/IL-17A, IL-17F, TNF-α, IL-6, IFN-γ, IL-1β ↓	Inhibit Hippo-YAP signaling pathway; inhibit IL-23/JAK2/STAT3 signaling pathway	Hepatocellular carcinoma model; Con A-induced acute liver injury model	[39,65]
IsoA	IL-1β, IL-6, TNF-α, NO ↓	Inhibit the NADPH oxidase/NF-κB pathway	LPS-induced mouse model of memory impairment	[66]
PhGs	IL-1β, TNF-α ↓	Inhibit activation of the NF-κB and JAK2 inflammatory signaling pathways	DSS-induced inflammatory bowel disease (IBD) model; patients with degenerative cervical myelopathy (DCM) after surgery	[67,68]
PhGs	IL-10 ↑	Regulate gut microbiota metabolism and restore immune homeostasis	DSS-induced inflammatory bowel disease (IBD) model	[67]
PhGs	MDA↓SOD & GSH-Px ↑	Enhance antioxidant enzyme activity, regulate gut microbiota and adipogenesis; protect synaptic plasticity (Alzheimer’s disease); activate Nrf2 pathway (neuroprotection)	Senescence-accelerated rat model; beta amyloid 1-42-induced Alzheimer’s disease rat model; high-fat diet-induced obesity model; MPTP-induced Parkinson’s disease mouse model	[69,70,71,72]

**Table 3:** Summary of combination/adjunct effect studies of phenylethanoid glycosides.

Comparison Components	Experimental Model	Combination/Adjunct Effects	Detection Method	References
Total Phenylethanoid Glycosides + Polysaccharides	SAMP6 mice (senescence-accelerated osteoporosis mice)	Prevented osteoporosis; ameliorated bone histopathological damage; promoted new bone, collagen fiber, chondrocyte formation and calcium deposit; improved bone microarchitecture; regulated bone turnover biomarkers (no synergy assessment performed)	Goldner’s Trichrome staining, Van Gieson (VG) staining, Safranin O-Fast Green staining, H&E staining, Von Kossa staining, micro-CT, tetracycline-calcein labeling, ELISA, Western blotting, immunohistochemistry	[16]
Echinacoside + Acteoside	Ovariectomized rats combined with D-galactose and AlCl_3_ (osteoporosis + Alzheimer’s disease concurrent model)	Improved learning and memory ability, protected hippocampal neurons; increased bone mineral density, inhibited bone resorption, improved bone microarchitecture and bone strength; showed estrogen-like effects (no synergy assessment performed)	Morris Water Maze test, ELISA, HE staining, Micro-CT, bone histomorphometry, biomechanical test	[72]

***Note:*** *Synergy is defined as the combined effect greater than the sum of individual effects, and only effects verified by combination index/isobologram/Bliss analysis are labeled as synergistic; others are referred to as combination/adjunct effects.*

**Table 4:** Summary table of multi-target regulatory signaling pathways and applications of phenylethanoid glycosides from *Cistanche deserticola* Y. C. Ma.

Name	Experimental Models	Core Biological Activities	Key Signaling Pathways/Targets	References
Echinacoside	Ovarian cancer model; liver cancer model; sports injury model; heart failure rat model; Parkinson’s disease model; UVB-induced skin injury model; inflammation model; Hirschsprung’s disease model.	Anti-inflammatory; immunomodulatory; anti-tumor; alleviation of sports injuries; hepatoprotective; neuroprotective; antioxidant; protection against UV-induced skin damage.	PI3K/AKT; miR-503-3p/TGF-β1/Smad; NADPH/ROS/ER stress pathway.	[40,41,44,45,49,52,73,74,80,82,85]
Acteoside	Allergic asthma model; NAFLD cell model; SH-SY5Y cell culture system; acute kidney injury (AKI) mouse model; experimental autoimmune encephalomyelitis model.	Immunomodulation; hepatoprotection and metabolic regulation; anti-tumor activity; antioxidation; neuroprotection; anti-inflammation.	AhR/CYP1A1; p38/Nrf2/HO-1; TREM2/PI3K/AKT signaling axis; ONOO^−^/mitochondrial autophagy pathway.	[37,45,60,61,62,80,82]
Isoacteoside	Liver cancer cell culture system; LPS-induced mouse AD model.	Anti-tumor activity; neuroprotection; inhibition of oxidative stress.	NADPH/NF-κB; PDHB (metabolic reprogramming).	[65,95]
Phenylethanoid glycosides	AD animal model; animal model of cognitive dysfunction; high-fat diet-induced obese mice; DSS-induced inflammatory bowel disease model	Neuroprotection and repair; immunomodulation; antioxidation and anti-inflammation; hepatoprotection and metabolic regulation.	SOD/CAT/GSH-Px pathway; PPAR-γ signaling pathway; AMPK-α; IRS1/AKT/GLUT4 signaling pathway.	[66,68,69,70,98,101]

## Data Availability

No data was used for the research described in this article.

## Abbreviations

*C. deserticola*, *Cistanche deserticola* Y. C. Ma; PhGs, Phenylethanoid Glycosides; ECH, Echinacoside; AS, Acteoside; Tub B, Tubuloside B; Cis A, Cistanoside A; Iso A, Isoacteoside; NO, nitric oxide; TNF-α, tumor necrosis factor-α; IL-6, interleukin-6; PBMCs, peripheral blood mononuclear cells; IBD, Inflammatory bowel disease; ROS, Reactive Oxygen Species; TLR4, Toll-like Receptor 4; AMPK, Adenosine Monophosphate-Activated Protein Kinase; SIRT1, sirtuin 1; NF-κB, nuclear factor-κB; MAPK, mitogen-activated protein kinase; IL-1β, interleukin-1β; Nrf2, nuclear factor erythroid 2-related factor 2; GBM, glioblastoma multiforme; HSCR, Hirschsprung’s Disease; AD, Alzheimer’s disease; HIRI, Hepatic ischemia–reperfusion injury; HMGB1, high mobility group box 1; LSECs, liver sinusoidal endothelial cells; IRF1, interferon regulatory factor 1; SASP, senescence-associated secretory phenotype; PCBP2, poly(rC)-binding protein 2; NAFLD, non-alcoholic fatty liver disease; HPA, hypothalamic–pituitary–adrenal; CTL, cytotoxic T lymphocyte; AhR, aryl hydrocarbon receptor; LPS, lipopolysaccharide; GSH-Px, glutathione peroxidase; PPAR-γ, peroxisome proliferator-activated receptor-γ; SARs, structure–activity relationships; STAT1, signal transducer and activator of transcription 1; cGAS-STING, cyclic GMP-AMP synthase-stimulator of interferon genes; PI3K/AKT/mTOR, phosphatidylinositol 3-kinase/protein kinase B/mammalian target of rapamycin.
